# Comparative phylogeography of two related plant species with overlapping ranges in Europe, and the potential effects of climate change on their intraspecific genetic diversity

**DOI:** 10.1186/1471-2148-11-29

**Published:** 2011-01-27

**Authors:** Gemma E Beatty, Jim Provan

**Affiliations:** 1 School of Biological Sciences, Queen's University Belfast, 97 Lisburn Road, Belfast BT9 7BL, Northern Ireland

## Abstract

**Background:**

The aim of the present study was to use a combined phylogeographic and species distribution modelling approach to compare the glacial histories of two plant species with overlapping distributions, *Orthilia secunda *(one-sided wintergreen) and *Monotropa hypopitys *(yellow bird's nest). Phylogeographic analysis was carried out to determine the distribution of genetic variation across the range of each species and to test whether both correspond to the "classic" model of high diversity in the south, with decreasing diversity at higher latitudes, or whether the cold-adapted *O. secunda *might retain more genetic variation in northern populations. In addition, projected species distributions based on a future climate scenario were modelled to assess how changes in the species ranges might impact on total intraspecific diversity in both cases.

**Results:**

Palaeodistribution modelling and phylogeographic analysis using multiple genetic markers (chloroplast *trn*S-*trn*G region, nuclear ITS and microsatellites for *O. secunda*; chloroplast *rps*2, nuclear ITS and microsatellites for *M. hypopitys*) indicated that both species persisted throughout the Last Glacial Maximum in southern refugia. For both species, the majority of the genetic diversity was concentrated in these southerly populations, whereas those in recolonized areas generally exhibited lower levels of diversity, particularly in *M. hypopitys*. Species distribution modelling based on projected future climate indicated substantial changes in the ranges of both species, with a loss of southern and central populations, and a potential northward expansion for the temperate *M. hypopitys*.

**Conclusions:**

Both *Orthilia secunda *and *Monotropa hypopitys *appear to have persisted through the LGM in Europe in southern refugia. The boreal *O. secunda*, however, has retained a larger proportion of its genetic diversity in more northerly populations outside these refugial areas than the temperate *M. hypopitys*. Given that future species distribution modelling suggests northern range shifts and loss of suitable habitat in the southern parts of the species' current distributions, extinction of genetically diverse rear edge populations could have a significant effect in the rangewide intraspecific diversity of both species, but particularly in *M. hypopitys*.

## Background

Paleoclimatic evidence indicates that the Earth's temperature has been continually changing over time [1-3]. The glacial and interglacial cycles that characterised the Quaternary period (*ca. *2.6 MYA - present) have had a significant effect on the distributions of species, particularly in the northern latitudes [4,5]. Temperate species were generally confined to low-latitude refugia throughout glacial periods and recolonized from these areas as the climate warmed during interglacials [6,7]. For plant species, however, whose spread is primarily via dispersal of seeds, the capacity to track changes in suitable habitat is limited, particularly for animal-dispersed species [8].

Understanding the past movements of species may help us understand how present and future climate change might affect species' ranges [9,10]. Within the last decade, it has become evident that anthropogenically induced climate change is causing shifts in the distribution ranges of many species [11-14]. As projections of carbon emissions suggest that this period of global warming will not end soon, these range shifts are likely to continue, but where species lack the migratory capacity to track changes in climate and available habitat, population extinctions may become increasingly frequent, particularly at species' low-latitude range edges [14-17]. Range-edge populations have generally been perceived as being genetically depauperate [18,19], although it has recently been suggested that some rear-edge populations may serve as reservoirs of unique genetic variation [20]. The processes of persistence in southern refugia during glacial maxima followed by northward recolonization have led to a pattern of "southern richness versus northern purity" [21-23], where the majority of genetic variation is found in populations that currently occupy previous refugial areas, with a northward decrease in genetic diversity due to progressive founder effects during the recolonization process (but see [24-27]). Consequently, if rear-edge populations are at particular risk of extinction due to the effects of climate change, their loss may have a disproportionally detrimental impact on overall levels of within-species genetic diversity, and such genetic erosion might compromise the long-term evolutionary potential of impacted species [28]. Assuming that species will shift their ranges north in response to global warming, genetically diverse southern edge populations of temperate species may be at the greatest risk of extinction, whereas cold-adapted species that might have persisted in more northerly refugia [24-27] could conceivably retain a larger proportion of their genetic diversity since this variation may not be concentrated in low latitude populations.

**Figure 1:**
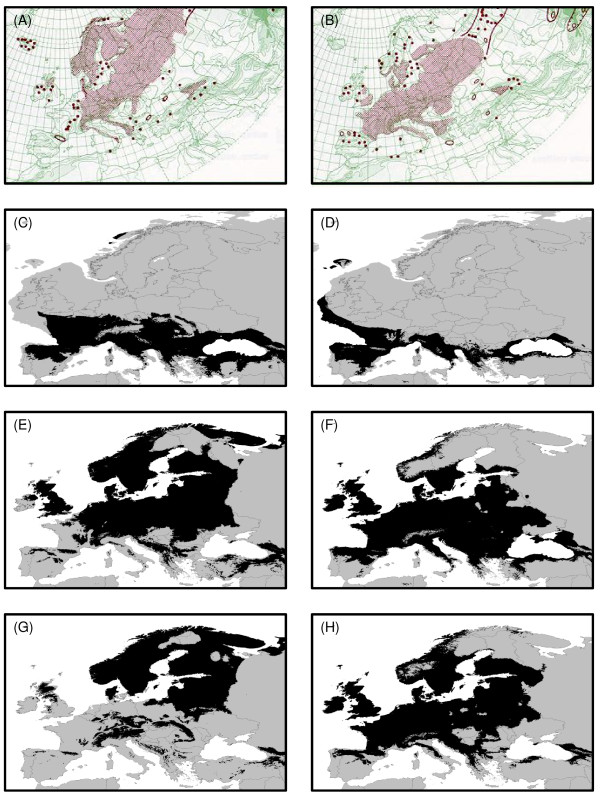
**Distributions of *O. secunda *and *M. hypopitys*, and modelled LGM, current and future distributions**. (A) Distribution of *O. secunda *(Source: Naturhistoriska riksmuseet) (B) Distribution of *M. hypopitys *(Source: Naturhistoriska riksmuseet) (C) Modelled LGM (*ca. *18 KYA) distribution of *O. secunda *(D) Modelled LGM (*ca. *18 KYA) distribution of *M. hypopitys *(E) Modelled current distribution of *O. secunda *(F) Modelled current distribution of *M. hypopitys *(G) Modelled future (2100) distribution of *O. secunda *(D) Modelled future (2100) distribution of *M. hypopitys*.

The aim of the present study was to use a combined phylogeographic and species distribution modelling approach to compare the glacial histories of two plant species, *Orthilia secunda *(one-sided wintergreen) and *Monotropa hypopitys *(yellow bird's nest). Both species belong to the Monotropoideae and have largely overlapping ranges in Europe (Figures 1A and 1B), as well as being found in North America, where they both exhibit disjunct east/west distributions. *O. secunda *is generally found in boreal forests, whereas *M. hypopitys *is usually associated with more temperate tree species and thus a comparison of the two should provide insights into the relative effects of climate change on a temperate species vs. a boreal species. Phylogeographic analysis was carried out to determine the distribution of genetic variation across the range of each species and to test whether both correspond to the "classic" model of high diversity in the south, with decreasing diversity at higher latitudes, or whether the cold-adapted *O. secunda *might retain more genetic variation in northern populations. In addition, projected species distributions based on a future climate scenario were modelled to assess how changes in the species ranges might impact on total intraspecific diversity in both cases.

## Methods

### Sampling and DNA extraction

Samples of *Orthilia secunda *and *Monotropa hypopitys *were obtained from 35 and 19 locations respectively throughout Europe (Tables 1 and 2). DNA was extracted using the Qiagen DNeasy kit. For *O. secunda*, 206 individuals were sequenced for the chloroplast *trn*S-*trn*G intergenic spacer, 154 individuals were sequenced for the nuclear internal transcribed spacer (ITS) region, and 218 individuals genotyped for five nuclear microsatellite loci. For *M. hypopitys*, 100 individuals were sequenced for part of the chloroplast *rps*2 gene, 100 individuals were sequenced for the nuclear ITS region, and 111 individuals were genotyped for eight nuclear microsatellite loci.

**Table 1:** *Orthilia secunda *populations analysed in this study

Country	Location	Code	Lat	Long	***N***_***cp***_	***N***_***ITS***_	***N***_***micro***_	Collector
Austria	Radmer an der Stube	ATRS	47.5556	14.7861	5	2	5	Apollonie Mayr
	Steiermark	ATS1	47.4967	14.3522	7	5	8	Peter Schönswetter
	Steiermark	ATS2	47.4389	14.9233	8	7	8	Peter Schönswetter
Czech Republic	Kosatky	CZKO	50.3178	14.6719	7	6	7	Petr Kotlik
Estonia	Jõgevamaa	EEJO	58.6338	26.9453	7	2	8	Teene Talve
	Nigula Nature Reserve	EENN	58.0194	24.6825	7	5	8	M. Reintal
	Põlvamaa	EEPO	58.0956	27.0302	8	4	8	T. Oja
France	Cervieres	FRCE	44.8667	6.7225	7	5	8	Rolland Douzet
	Sauvas	FRSA	44.6004	5.9037	5	4	7	Arne Saatkamp
	Station Alpine Joseph Fourier	FRJF	45.0360	6.4002	6	5	8	Rolland Douzet
Ireland	Correl Glen	IECG	54.4372	-7.8744	4	4	4	Gemma Beatty
	Cranny Burn	IECB	54.9114	-6.0409	4	4	4	Gemma Beatty
Italy	Valle D'Aosta	ITVA	45.7125	7.1639	6	5	6	Nationaal Herbarium Nederland
Montenegro	Durmitor Mountains	MEDM	43.1611	19.2028	8	7	8	Anna & Michal Ronikier
	Komovi Massif	MEKM	42.6947	19.6672	5	4	5	Anna & Michal Ronikier
Norway	Buskerud	NOBU	60.1208	10.3833	8	6	8	Andreas Tribsch
	Oslo	NOOS	59.9939	10.7064	8	6	8	Andreas Tribsch
	Selvikstaken	NOSE	58.8625	6.0750	4	4	4	Andreas Tribsch
	Troms Fylke	NOTF	68.9500	19.7500	4	4	5	W. Paul
Poland	Bialystok	PLBI	53.1167	23.1167	8	4	8	Ada Wroblewska
	Kielce	PLKI	50.8400	20.5800	5	5	5	W. Paul
	Pomorze Zachodnie	PLPZ	54.0047	19.9983	7	6	8	Joanna Julia & Lech Galosz
Scotland	Glen Glass	SCGG	57.6816	-4.4226	4	4	4	Peter McEvoy
	Glen Mhor	SCGM	56.8844	-3.6315	4	4	4	Peter McEvoy
Slovakia	Muranska Planina	SKMP	48.7825	19.9600	8	5	8	Anna & Michal Ronikier
	Nizke Tatry	SKNT	48.9983	19.5875	8	5	8	Anna & Michal Ronikier
	Slovensky Raj	SKSR	48.9305	20.2897	2	2	2	Anna & Michal Ronikier
	Zapadne Tatry	SKZT	49.1453	19.7850	7	6	7	Anna & Michal Ronikier
Slovenia	Kaminske Alpe	SIKA	46.3922	14.6000	8	6	8	Peter Schönswetter
Sweden	Flurkmark	SEFL	64.1273	20.1322	8	7	8	Stefan Ericsson
	Lomselenas	SELO	65.1441	17.3139	8	6	8	Stefan Ericsson
	Ranas	SERA	59.8128	18.2883	5	1	8	Arne Anderberg
Switzerland	Chasseron	CHCH	46.8287	6.5508	6	6	6	Philippe Druart
	Valais	CHVA	46.0000	7.6833	5	5	5	Nationaal Herbarium Nederland
					206	154	218	

### Species distribution modelling

Ecological niche modelling (ENM) was carried out to determine suitable climate envelopes for *O. secunda *and *M. hypopitys *in Europe for the LGM (*ca. *18KYA), and the year 2100 under a future climate scenario using the maximum entropy approach implemented in the MAXENT software package (V3.2.1; [29]). Species occurrence data were downloaded from the Global Biodiversity Information Facility data portal (http://www.gbif.org), totalling 14,221 and 8,829 occurrences for *O. secunda *and *M. hypopitys *respectively. A principal component analysis (PCA) was carried out on the 19 BIOCLIM variables in the WorldClim data set [30] to remove correlated variables, since these can lead to overfitting of the model. After removing variables that exhibited a strong correlation (Spearman's rank correlation >0.5; [31]), three variables (P1 [Annual Mean Temperature], P4 [Temperature Seasonality] and P14 [Precipitation of Driest Period]) were used to generate ENMs at 2.5 minute resolution using MAXENT with the default parameters for convergence threshold (10^-5^) and number of iterations (500), and projected onto reconstructed LGM data (Community Climate System Model [CCSM]; Palaeoclimate Modelling Intercomparison Project Phase II: http://pmip2.lsce.ipsl.fr) to identify potential refugial areas. The current climate envelope was also projected onto climate grids corresponding to the same three bioclimatic variables in the year 2100 under the National Centre for Atmospheric Research general circulation model (CCM3 model) that simulates double CO_2 _emissions [32]. Duplicate records from the same locality were removed to reduce the effects of spatial autocorrelation. Presence thresholds were determined using the sensitivity-specificity sum maximisation approach [33] and the performance of the models were tested using 25% of the occurrence data points to determine the area under the receiver operating characteristic (ROC) curve (AUC).

### Molecular genetic analyses - *O. secunda*

206 individuals were sequenced for the chloroplast *trn*S-*trn*G intergenic spacer. A product was amplified using the *O. secunda*-specific primers and reaction conditions described in [34]. 5 μl PCR product were resolved on 1.5% agarose gels and visualised by ethidium bromide staining, and the remaining 15 μl sequenced in both directions using the BigDye sequencing kit (V3.1; Applied Biosystems) and run on an AB 3730XL DNA analyser.

154 individuals were sequenced for a section of the nuclear ITS region. Primers were designed from GenBank sequence accession number AF133747: OS-ITS-F 5'-TGTTTGTACACTTGGGGAAGC-3' and OS-ITS-R 5'-TCGCGGTCAATGTACCGTAG-3'. PCR and sequencing were carried out as described in [34], except that an annealing temperature of 55°C was used for the PCR.

218 individuals were genotyped for five *O. secunda *microsatellite loci previously described in [35]. Forward primers were modified by the addition of a 19 bp M13 tail (5'-CACGACGTTGTAAAACGAC-3') and reverse primers were modified by the addition of a 7 bp tail (5'-GTGTCTT-3'). PCR was carried out in a total volume of 10 μl containing 100 ng genomic DNA, 10 pmol of dye-labelled M13 primer (6-FAM or HEX), 1 pmol of tailed forward primer, 10 pmol reverse primer, 1× PCR reaction buffer, 200 μM each dNTP, 2.5 mM MgCl_2 _and 0.25 U GoTaq Flexi DNA polymerase (Promega). PCR was carried out on a MWG Primus thermal cycler using the conditions described in [35] and genotyping was carried out on an AB3730xl capillary genotyping system. Allele sizes were scored in GENEMAPPER V4.1 using ROX-500 size standards and were checked by comparison with previously sized control samples.

### Molecular genetic analyses - *M. hypopitys*

100 individuals were sequenced for a section of the chloroplast *rps*2 gene. Primers were designed from GenBank sequence accession number AF351956 (Bidartondo and Bruns 2001): MH-rps2-F 5'-TTCGCCGATTTAGTATCACG-3' and MH-rps2-R 5'-GGGATTCCCAAAGTAATACATTCTA-3'. PCR and sequencing were carried out as described in [34].

100 individuals were sequenced for a section of the nuclear ITS region. Primers were designed from GenBank sequence accession number AF384126[36]: MH-ITS-F 5'-GGTTGGCCTACCCTTTATTTT-3' and MH-ITS-R 5'-GAAGTAATCCAATCATAACACTGACA-3'. PCR and sequencing were carried out as described in [34], except that an annealing temperature of 55°C was used.

111 individuals were genotyped for five *M. hypopitys *microsatellite loci previously described in [37] - Mono02, Mono15, Mono20, Mono21 and Mono22. Three additional loci developed using the ISSR-cloning technique outlined in [38] were also used (Table 2). Forward primers were modified by the addition of a 19 bp M13 tail (5'-CACGACGTTGTAAAACGAC-3') and reverse primers were modified by the addition of a 7 bp tail (5'-GTGTCTT-3'). PCR was carried out in a total volume of 10 μl containing 100 ng genomic DNA, 10 pmol of dye-labelled M13 primer (6-FAM or HEX), 1 pmol of tailed forward primer, 10 pmol reverse primer, 1× PCR reaction buffer, 200 μM each dNTP, 2.5 mM MgCl_2 _and 0.25 U GoTaq Flexi DNA polymerase (Promega). PCR was carried out on a MWG Primus thermal cycler using the conditions described in [39] and genotyping was carried out on an AB3730xl capillary genotyping system. Allele sizes were scored in GENEMAPPER V4.1 (Applied Biosystems) using ROX-500 size standards and were checked by comparison with previously sized control samples.

**Table 2:** *Monotropa hypopitys *populations analysed in this study

Country	Location	Code	Lat	Long	***N***_***cp***_	***N***_***ITS***_	***N***_***micro***_	Collector
Austria	Karnten	ATKA	46.5228	13.9539	2	2	2	Peter Schönswetter
Czech Republic	Polom	CZPO	49.7892	15.7595	1	1	1	Jakub Tiesetel
England	Peasmarsh	ENPE	50.9667	-0.6667	6	6	8	Jonathan Simmons
Estonia	Jõgevamaa	EEJO	58.6338	26.9453	6	6	8	Teene Talve
	Põlvamaa	EEPO	58.0956	27.0302	7	8	8	T. Ota
Ireland	Ely Lodge	IEEL	54.4567	-7.9002	8	7	8	Gemma Beatty
	Straidkilly	IEST	54.9914	-6.0409	8	7	8	Gemma Beatty
Poland	Czarne Lake	PLCL	53.4667	20.6000	8	7	8	Ada Wroblewska
	Lake Golun	PLLG	54.0047	17.9983	8	8	8	Ada Wroblewska
	Knyszyn	PLKN	53.3333	22.9167	8	8	8	Joanna Julia & Lech Galosz
Romania	Retezat Mountains	RORM	45.3097	22.9678	8	8	8	Anna & Michal Ronikier
		ROVG	46.2070	25.5400	4	4	6	Anna Maria Csergo
Slovakia	Muranska Planina	SKMP	48.7825	19.9600	2	2	2	Anna & Michal Ronikier
	Nizke Tatry	SKNT	48.9983	19.5875	4	4	6	Anna & Michal Ronikier
Slovenia	Dolenjska	SIDO	45.9236	15.0958	2	3	3	Peter Schönswetter
	Soca Valley	SISV	46.3450	13.6800	8	8	8	Peter Schönswetter
Sweden	Ranas	SERA	59.8128	18.2883	3	4	4	Arne Anderberg
Switzerland	Chasseron	CHCH	46.8287	6.5508	5	5	5	Philippe Druart
					100	100	111	

### Data analysis

Alignments were constructed using BIOEDIT (V7.0.9.0) [40] for the *O. secunda *chloroplast *trn*S-*trn*G intergenic spacer and nuclear ITS, and for the *M. hypopitys *chloroplast *rps*2 and nuclear ITS. Length variation at any mononucleotide repeat regions was removed, since the bidirectional mutation model operating at such regions can give rise to homoplasy [41]. The alignments were used to construct statistical parsimony networks using the TCS software package (V1.2.1) [42]. Where reticulations were present in the networks, these were broken following the rules described in [43].

Tests for linkage disequilibrium between pairs of microsatellite loci in each population were carried out in the program FSTAT [44]. Levels of genetic diversity were calculated for populations with a sample size of *N ≥ *4. Gene diversity (*H*) based on haplotype frequencies for the *O. secunda *chloroplast *trn*S-*trn*G region and nuclear ITS, and the *M. hypopitys *chloroplast *rps*2 and nuclear ITS, and observed and expected heterozygosity (*H*_*O *_and *H*_*E*_) based on nuclear microsatellite allele frequencies were calculated using the ARLEQUIN software package (V3.01) [45]. Population structuring based on the microsatellite data was determined using the STRUCTURE software package (V 2.2) [46]. Five independent runs were carried out for all values of *K*, the number of clusters, between 2 and 20. The program was run each time using 50,000 burn-in iterations followed by 500,000 Markov Chain Monte Carlo iterations, and the most likely value of *K *was determined using the *ΔK *statistic [47].

## Results

### Species distribution modelling

For all models, the area under the receiver operating curve (AUC) statistic was consistently higher than 0.95, indicating good performance.

Distribution modelling for *O. secunda *and *M. hypopitys *at the LGM indicated extensive areas of suitable habitat for both species in southern Europe (Figures 1C and 1D). For *O. secunda*, two of the French populations (FRSA and FRCE), one of the Swiss populations (CHVA) and the two populations from Montenegro lay within the suitable climate envelope indicated by the ENM. None of the *M. hypopitys *populations studied lay within the suitable climate envelope indicated by the ENM.

The future distribution model indicated an extensive loss of suitable habitat for *O. secunda *relative to the modelled current climate envelope (Figure 1E), particularly in northern central Europe (Figure 1G). The majority of the suitable remaining habitat in southern Europe would be largely restricted to the mountainous regions of the Pyrenees, the Alps, the Carpathians and the Dinaric Alps. For *M. hypopitys*, the model indicated a general northward shift in the species' distribution, with a loss of suitable habitat in southeastern Europe but an increase in northern Europe, particularly in Scandinavia (Figures 1F and 1H).

### *O. secunda *phylogeography

**Figure 2:**
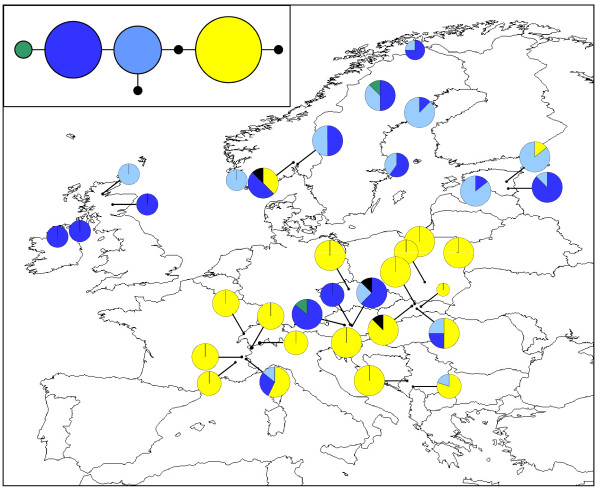
**Geographical distribution of *O. secunda *chloroplast *trn*S-*trn*G haplotypes**. Pie chart sizes are approximately proportional to sample size, with the smallest circles representing *N *= 1 and the largest representing *N *= 8. Inset shows the phylogenetic relationships between the seven haplotypes. Small black circles represent unique haplotypes i.e. those found in a single individual. The population of origin of each unique haplotype is indicated.

Removal of length polymorphism at three mononucleotide repeat regions from the chloroplast *trn*S-*trn*G alignment resulted in an overall alignment length of 495 bp and seven distinct haplotypes (Table 3; Figure 2; GenBank sequence accession numbers HQ864688-HQ864694). Three of these (Haplotypes 5, 6 and 7) were unique to a single individual. The three most common haplotypes exhibited a general north-south split, with the Haplotype 2 (yellow) found predominantly in southern populations whilst northern populations contained primarily the two blue haplotypes (Haplotypes 1 and 3). Two populations contained all three of these haplotypes: the FRCE population (France) and the SKMP population (Slovakia). The fourth non-unique haplotype, Haplotype 4 (green), was found in a single individual in both the ATST1 (Austria) and the SELO (Sweden) populations.

**Table 3:** Diversity statistics for *O. secunda *populations

Country	Code	***H***_***E***_	cpDNA haplotype							ITS haplotype				
			
			1	2	3	4	5	6	7	1	2	3	4	5
Austria	ATRS	0.729	-	5	-	-	-	-	-	5	-	-	-	-
	ATS1	0.529	-	6	-	1	-	-	-	7	-	-	-	-
	ATS2	0.629	-	5	2	-	1	-	-	8	-	-	-	-
Czech Republic	CZKO	0.736	7	-	-	-	-	-	-	4	2	-	-	-
Estonia	EEJO	0.768	1	-	6	-	-	-	-	1	-	-	-	-
	EENN	0.752	-	1	6	-	-	-	-	-	-	5	-	-
	EEPO	0.797	-	7	1	-	-	-	-	1	-	-	-	-
France	FRCE	0.737	4	2	1	-	-	-	-	3	1	-	-	1
	FRSA	0.811	5	-	-	-	-	-	-	2	-	-	2	-
	FRJF	0.765	6	-	-	-	-	-	-	5	-	-	-	-
Ireland	IECG	0.400	-	4	-	-	-	-	-	4	-	-	-	-
	IECB	0.643	-	4	-	-	-	-	-	4	-	-	-	-
Italy	ITVA	0.637	6	-	-	-	-	-	-	5	-	-	-	-
Montenegro	MEDM	0.757	8	-	-	-	-	-	-	7	-	-	-	-
	MEKM	0.807	4	-	1	-	-	-	-	4	-	-	-	-
Norway	NOBU	0.727	3	4	-	-	-	1	-	6	-	-	-	-
	NOOS	0.839	-	4	4	-	-	-	-	5	-	-	1	-
	NOSE	0.839	-	-	4	-	-	-	-	4	-	-	-	-
	NOTF	0.409	-	3	1	-	-	-	-	4	-	-	-	-
Poland	PLBI	0.493	8	-	-	-	-	-	-	4	-	-	-	-
	PLKI	0.582	5	-	-	-	-	-	-	5	-	-	-	-
	PLPZ	0.770	7	-	-	-	-	-	-	6	-	-	-	-
Scotland	SCGG	0.429	-	-	4	-	-	-	-	4	-	-	-	-
	SCGM	0.529	-	4	-	-	-	-	-	4	-	-	-	-
Slovakia	SKMP	0.807	4	2	2	-	-	-	-	4	1	-	-	-
	SKNT	0.772	7	-	-	-	-	-	1	5	-	-	-	-
	SKSR	NC	2	-	-	-	-	-	-	2	-	-	-	-
	SKZT	0.763	7	-	-	-	-	-	-	6	-	-	-	-
Slovenia	SIKA	0.755	8	-	-	-	-	-	-	4	2	-	-	-
Sweden	SEFL	0.517	-	4	3	1	-	-	-	7	-	-	-	-
	SELO	0.435	-	1	7	-	-	-	-	6	-	-	-	-
	SERA	0.735	-	3	2	-	-	-	-	1	-	-	-	-
Switzerland	CHCH	0.673	6	-	-	-	-	-	-	6	-	-	-	-
	CHVA	0.755	5	-	-	-	-	-	-	5	-	-	-	-

**Figure 3:**
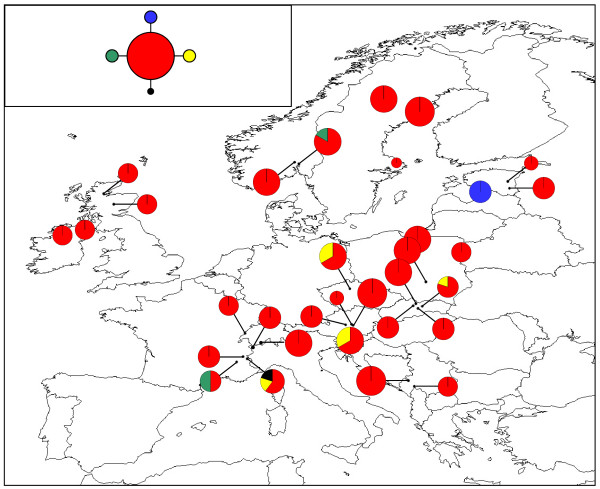
**Geographical distribution of *O. secunda *nuclear ITS haplotypes**. Pie chart sizes are approximately proportional to sample size, with the smallest circles representing *N *= 1 and the largest representing *N *= 8. Inset shows the phylogenetic relationships between the five haplotypes.

The 475 bp nuclear ITS alignment contained five distinct haplotypes (Table 3; Figure 3; GenBank sequence accession numbers HQ864695-HQ864699). The most common haplotype, Haplotype 1 (red), was found in all populations with the exception of the EENN population (Estonia). Only six populations exhibited any within-population variation (FRCE, FRSA [both France], SIKA [Slovenia], SKMP [Slovakia], CZKO [Czech Republic] and NOOS [Norway]) and only the FRCE population contained more than two haplotypes. The EENN population was fixed for Haplotype 3 (blue), which was not found elsewhere.

**Figure 4:**
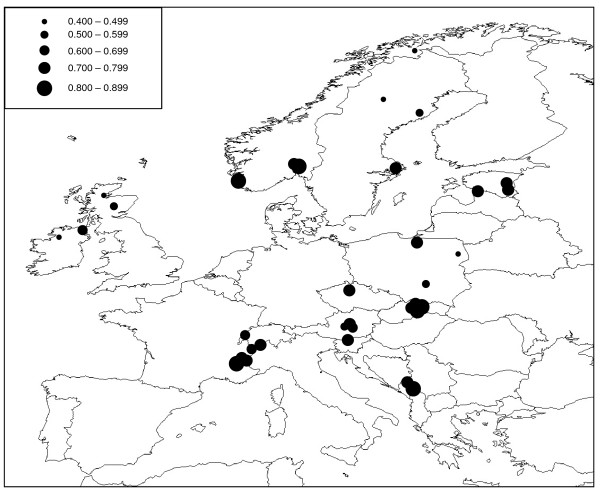
**Expected heterozygosity (*H***_***E***_**) in *O. secunda *populations based on five nuclear microsatellite loci**. Circle sizes are indicative of level of *H*_*E *_(see inset).

**Figure 5:**
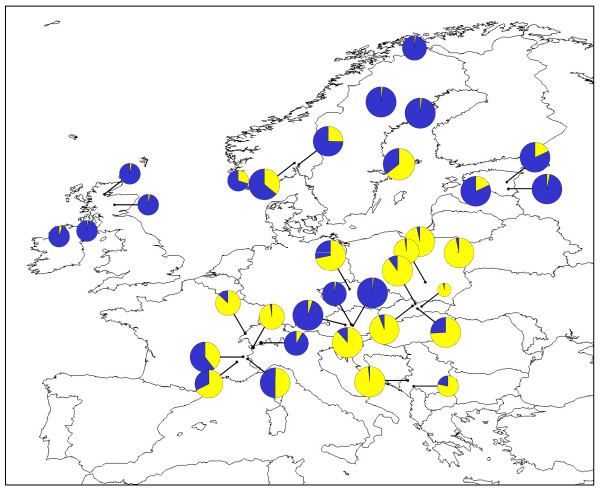
**Assignment of *O. secunda *populations to *K *= 2 clusters based on STRUCTURE analysis of the nuclear microsatellite data**.

No significant linkage disequilibrium was detected between pairs of microsatellite loci after sequential Bonferroni correction. Between 16 and 30 alleles were detected at the five loci studied (mean = 20.20) and levels of expected heterozygosity (*H*_*E*_) calculated for populations with a sample size of *N ≥ *4 ranged from 0.400 (IECG [Ireland]) to 0.839 (NOOS and NOSE [both Norway]), with a mean value of 0.677 (Table 3; Figure 4). The STRUCTURE analysis of the microsatellite data indicated that the most likely number of genetic clusters was *K *= 2 (Figure 5).

### *M. hypopitys *phylogeography

**Figure 6:**
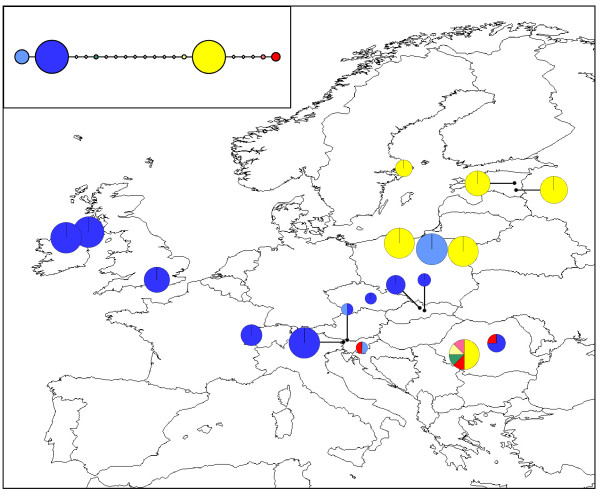
**Geographical distribution of *M. hypopitys *chloroplast *rps*2 haplotypes**. Pie chart sizes are approximately proportional to sample size, with the smallest circles representing *N *= 1 and the largest representing *N *= 8. Inset shows the phylogenetic relationships between the eight haplotypes. Open diamonds represent missing haplotypes.

The 320 bp chloroplast *rps*2 alignment contained seven distinct haplotypes (Table 4; Figure 6; GenBank sequence accession numbers HQ864700-HQ864706). The two most common haplotypes, Haplotypes 1 and 2 (depicted in blue and yellow), exhibited a largely east-west split. Only four populations exhibited any within-population variation (ATKA [Austria], SIDO [Slovenia], RORM and ROVG [both Romania]) and of these, only the RORM population contained more than two haplotypes.

**Table 4:** Diversity statistics for *M. hypopitys *populations

Country	Code	***H***_***E***_	cpDNA haplotype							ITS haplotype		
			
			1	2	3	4	5	6	7	1	2	3
Austria	ATKA	NC	1	-	1	-	-	-	-	-	2	-
Czech Republic	CZPO	NC	1	-	-	-	-	-	-	1	-	-
England	ENPE	0.624	-	6	-	-	-	-	-	6	-	-
Estonia	EEJO	0.690	-	6	-	-	-	-	-	-	6	-
	EEPO	0.573	7	-	-	-	-	-	-	-	8	-
Ireland	IEEL	0.500	8	-	-	-	-	-	-	7	-	-
	IEST	0.370	8	-	-	-	-	-	-	7	-	-
Poland	PLCL	0.516	-	-	8	-	-	-	-	7	-	-
	PLLG	0.716	-	8	-	-	-	-	-	-	8	-
	PLKN	0.740	-	8	-	-	-	-	-	-	8	-
Romania	RORM	0.731	-	4	-	1	1	1	1	-	8	-
	ROVG	0.710	3	-	-	1	-	-	-	3	1	-
Slovakia	SKMP	NC	2	-	-	-	-	-	-	2	-	-
	SKNT	0.682	4	-	-	-	-	-	-	3	1	-
Slovenia	SIDO	NC	-	-	1	1	-	-	-	1	1	1
	SISV	0.530	8	-	-	-	-	-	-	8	-	-
Sweden	SERA	0.674	-	3	-	-	-	-	-	4	-	-
Switzerland	CHCH	0.750	5	-	-	-	-	-	-	4	-	1

**Figure 7:**
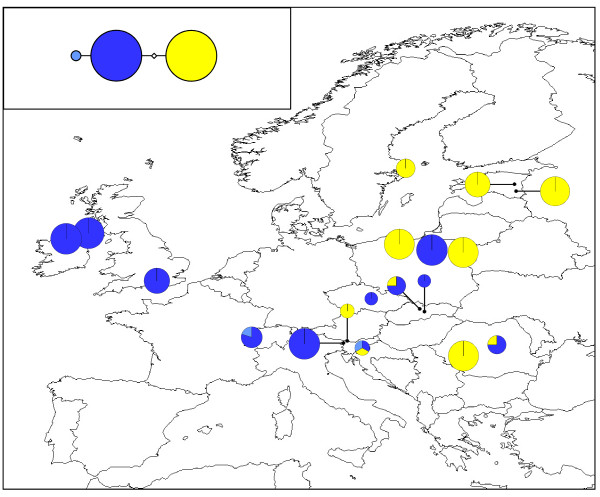
**Geographical distribution of *M. hypopitys *nuclear ITS haplotypes**. Pie chart sizes are approximately proportional to sample size, with the smallest circles representing *N *= 1 and the largest representing *N *= 8. Inset shows the phylogenetic relationships between the three haplotypes. Open diamonds represent missing haplotypes.

The 287 bp nuclear ITS alignment contained three distinct haplotypes (Table 4; Figure 7; GenBank sequence accession numbers HQ864707-HQ865709). The distribution of these haplotypes was broadly congruent with that of the chloroplast *rps*2 haplotypes. Only the CHCH (Switzerland), SIDO (Slovenia), SKNT (Slovakia) and ROVG (Romania) populations exhibited any within-population variation, with all three haplotypes being found in the SIDO population.

**Figure 8:**
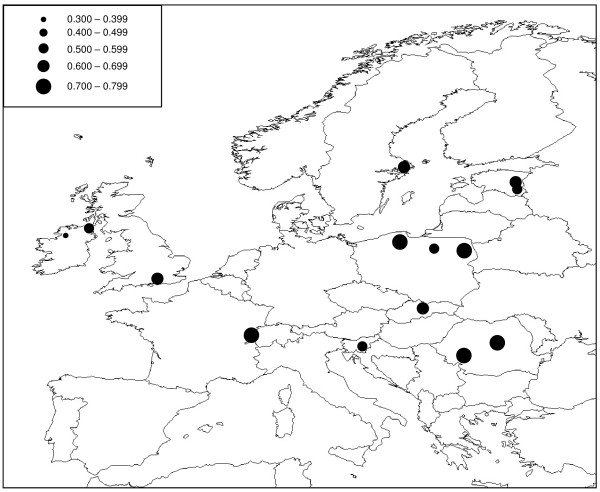
**Expected heterozygosity (*H***_***E***_**) in *M. hypopitys *populations based on five nuclear microsatellite loci**. Circle sizes are indicative of level of *H*_*E *_(see inset).

**Figure 9:**
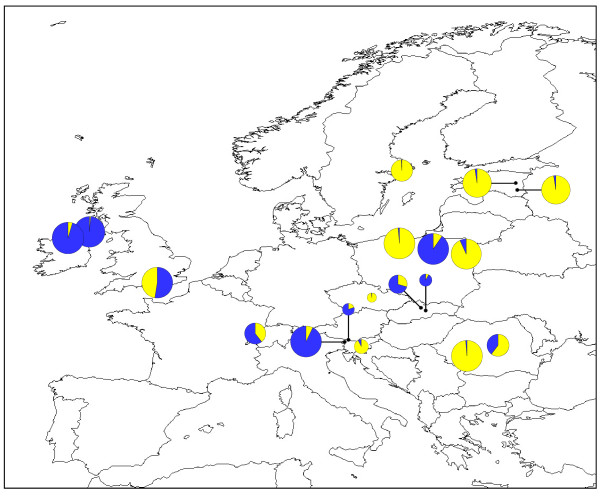
**Assignment of *M. hypopitys *populations to *K *= 2 clusters based on STRUCTURE analysis of the nuclear microsatellite data**.

No significant linkage disequilibrium was detected between pairs of microsatellite loci after sequential Bonferroni correction. Between 10 and 22 alleles were detected at the eight loci studied (mean = 15.125) and levels of expected heterozygosity (*H*_*E*_) calculated for populations with a sample size of *N ≥ *4 ranged from 0.370 (IEST [Ireland]) to 0.750 (CHCH [Switzerland]), with a mean value of 0.629 (Table 4; Figure 8). The STRUCTURE analysis of the microsatellite data indicated that the most likely number of genetic clusters was *K *= 2 (Figure 9).

## Discussion

It is now apparent that phylogeographic inferences based on a single, non-recombining marker can be misleading [48,49]. Consequently, phylogeographic studies are increasingly using multiple genetic markers and/or palaeodistribution modelling to draw more reliable inferences on population history. The results of the paleodistribution modelling and the patterns of genetic variation revealed by the phylogeographic analyses suggest that both *Orthilia secunda *and *Monotropa hypopitys *persisted throughout the LGM in Europe in southern refugia. Although both species generally exhibited a "southern richness vs. northern purity" distribution of genetic variation [21], this was more pronounced in the temperate *M. hypopitys*, where the only populations that displayed any within-population genetic variation for both the chloroplast *rps*2 and nuclear ITS regions were located closest to the modelled refugial areas. Northern populations of *O. secunda *were more diverse, but the signatures of refugial areas i.e. high diversity coupled with unique haplotypes [27] were restricted to southern populations.

Based on the weight of evidence across modelling and the different markers used, our findings indicate a possible refugial area for *O. secunda *in Europe located in the vicinity of the French Alps. A second area of high diversity and endemic haplotypes included the Austrian Alps and Slovakia, but these populations lie outside the suitable climate envelope indicated by the palaeodistribution model. Nevertheless, although the precise locations of putative refugia are difficult to identify accurately, it is clear that the majority of genetic diversity is contained in southern populations. The occurrence of a fixed endemic ITS haplotype in one of the Estonian populations (EENN) more likely represents a relatively recent mutation that has become fixed through genetic drift, rather than indicating an extreme northern refugium. For *M. hypopitys*, the modelling and genetic data both indicated a likely refugial area in southeastern Europe. The identification of two genetic clusters with a broadly northern/eastern vs. southern/western geographical distribution for both species based on microsatellite data could indicate isolation in separate refugia followed by differential recolonization after the retreat of the ice [24].

Many studies have used modelling approaches to determine the effects of present and future climate change on the distribution ranges of plant species (e.g. [50-52]). We can extend this approach to investigate the potential effects of such distribution changes on intraspecific genetic diversity. The future modelled distributions of both *O. secunda *and *M. hypopitys *indicate substantial changes in the ranges of both species. For *M. hypopitys *in particular, these changes could have a profound impact on the genetic diversity of the species in Europe. Previous studies have suggested that range contraction during previous phases of climate change was characterized by population extinction, rather than migration [6,53]. Although the future model indicates a range expansion at the northern edge, it also suggests extensive loss of suitable habitat in southeastern Europe. Given that this area represents the centre of genetic diversity for the species, extinction of these populations would lead to massive loss of genetic diversity since more northerly populations are genetically depauperate relative to populations in the southeast. A northern expansion of the species' range would not counter this, because the leading edge colonization would be from these low-diversity northern populations. Northern populations of *O. secunda*, however, tended to be more genetically diverse than those of *M. hypopitys*. Consequently, the loss of southern and central European *O. secunda *populations indicated by the species distribution model would not have the same overall effect on total intraspecific genetic diversity across the continent. Nevertheless, although the populations from the species' centres of diversity in the French and Austrian Alps would still lie within the future modelled climate envelope, this would most likely be as a result of altitudinal migration, since the mountain ranges of southern and eastern Europe represent the only climatically suitable areas in the region. Whilst altitudinal migration offers some short-term potential for countering the effects of climate change [54-57], its scope is ultimately limited [58]. The situation in Europe is somewhat different from that in North America, where the occurrence of northern refugia for both species means that a lower proportion of the total genetic diversity in the continent is concentrated in southern populations [[34], Beatty & Provan, unpublished results] and thus the impact of loss of rear-edge populations might not be as extreme. It should also be borne in mind that models of future (and, indeed, past) climate are not guaranteed to be 100% accurate, and that many other factors such as changes in species tolerances through adaptation and species-species interactions will also determine species current and future ranges. Nevertheless, at least in Europe, the adverse encroachment of human activity on the boreal and temperate woodlands that form the natural habitat for these species, coupled with the fact that climate is changing faster now than at any time in the past, means that the impacts on the gene pools and subsequent adaptive potential of these, and possibly many other species, are likely to be potentially serious.

## Conclusions

Both *Orthilia secunda *and *Monotropa hypopitys *appear to have persisted through the LGM in Europe in southern refugia. The boreal *O. secunda*, however, has retained a larger proportion of its genetic diversity in more northerly populations outside these refugial areas than the temperate *M. hypopitys*. Given that future species distribution modelling suggests northern range shifts and loss of suitable habitat in the southern parts of the species' current distributions, extinction of genetically diverse rear edge populations could have a significant effect in the rangewide intraspecific diversity of both species, but particularly in *M. hypopitys*.

## Authors' contributions

Both authors conceived and designed the study. GEB carried out the laboratory work. Both authors analysed the data and wrote the manuscript.
